# Missing Link Prediction using Common Neighbor and Centrality based Parameterized Algorithm

**DOI:** 10.1038/s41598-019-57304-y

**Published:** 2020-01-15

**Authors:** Iftikhar Ahmad, Muhammad Usman Akhtar, Salma Noor, Ambreen Shahnaz

**Affiliations:** 1 Department of Computer Science and Information Technology, University of Engineering and Technology, Peshawar, Pakistan; 2 Department of Computer Science, Shaheed Benazir Bhutto Woman University, Peshawar, Pakistan

**Keywords:** Computer science, Information technology

## Abstract

Real world complex networks are indirect representation of complex systems. They grow over time. These networks are fragmented and raucous in practice. An important concern about complex network is link prediction. Link prediction aims to determine the possibility of probable edges. The link prediction demand is often spotted in social networks for recommending new friends, and, in recommender systems for recommending new items (movies, gadgets etc) based on earlier shopping history. In this work, we propose a new link prediction algorithm namely “Common Neighbor and Centrality based Parameterized Algorithm” (CCPA) to suggest the formation of new links in complex networks. Using *AUC* (Area Under the receiver operating characteristic Curve) as evaluation criterion, we perform an extensive experimental evaluation of our proposed algorithm on eight real world data sets, and against eight benchmark algorithms. The results validate the improved performance of our proposed algorithm.

## Introduction

Complex networks are effective descriptions of real world networks, where real world problems can be modeled in the form of complex network graphs^1^. Complex networks describe the interaction among the elements of complex systems such as computer, neural, chemical and online social networks^2^. In such networks, entities (such as computer, neurons etc.) are represented by nodes (also called vertices), whereas edges between pair of nodes depict interactions/associations between the nodes^3^. Complex networks have application in many divisions of applied science^4^. It has been applied in health care to predict the spread of epidemic diseases^5^, and in the development of strategies to vaccinate the potential affectees to limit the spread of epidemic. Furthermore, the complex network analysis can be applied in legislative drives to influence maximum number of citizens^6^, and in the development of road networks to improve routes^7^. Considerable efforts are made to understand the network evolution^8,9^, and the fundamental topological structure of complex real world networks^10^.

Due to ever evolving nature of complex networks, one crucial scientific issue related to complex network analysis is missing link prediction^11^. Networks are very agile in nature; fresh vertices and edges are added over the passage of time^12^. The basic idea of link prediction is to approximate the possibility of the existence of a link between pair of nodes, derived from the current topological structural attributes of the nodes^13^. For example, in online connected community networks, future associations can be suggested as likely-looking friendships, which can assist the system in recommending new friends and thus strengthen their dependability to the service^8^. In other words, link prediction provides a measure of social propinquity between pair of nodes. The only available information is the topological structure of the network^14^.

Applications of the phenomenon include suggestion of new followers/friends on social websites such as Google Plus, Facebook, Foursquare, LinkedIn, and Twitter etc. In addition, it can also be used to suggest interests that are most likely collective. For example, recommendation of products on Amazon and Alibaba, recommendation of movies on Netflix, and ads display to users on Google AdWords and Facebook^15^.

In this work, we present a novel algorithm for link prediction using the existing topological structure of the network. Our proposed algorithm named *Common Neighbor and Centrality based Parameterized Algorithm* (CCPA) identifies potential future edges/connections between nodes using common neighbors and centrality. The proposed algorithm is parameterized, i.e., it has the flexibility to let the user/system set the importance of common neighbor and centrality. The proposed algorithm is evaluated against eight commonly used standard algorithms for link prediction on eight data sets. Experimental evaluation suggests the better predictability of our proposed algorithm.

## Formal Problem Setting

Assume *G*(*V*, *E*) to be an undirected graph, representing a complex network at a time *t*; *V* and *E* are building blocks of graph representing set of nodes and edges respectively. Loops and multiple-connections between nodes are not allowed^15^.

Let, *U* represents the set of all possible edges between nodes in the graph, then $|U|=\frac{|V|(|V|-1)}{2}$. Let, *L* = *U* − *E* be the set of missing links in the graph. Normally we are not aware which links may occur in the future, otherwise we do not need link prediction^16^. Link prediction aims to predict the possibility of link formation between two nodes at time *t*′ (*t*′ > *t*)^13^. The primary goal is to forecast new links among nodes that may take place in the near future.

**Figure 1:**
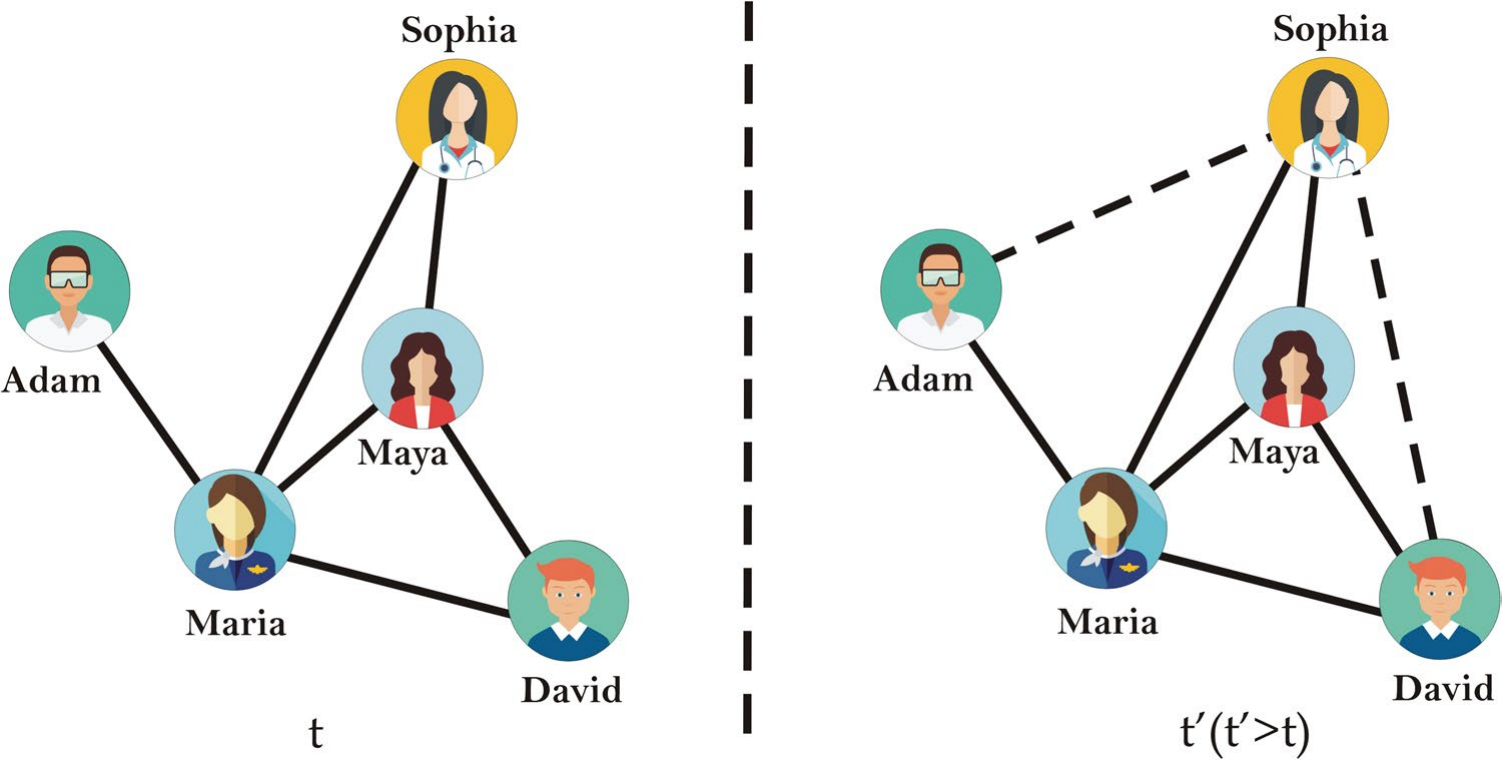
Graphical representation of missing link prediction; dashed lines depict possible edges.

The problem is clarified with a simple network of 5 persons (nodes) as shown in Fig. 1. The total number of possible links in a 5 node network is $\frac{5(5-1)}{2}=10$. For the missing links *L* = *U* − *E*, the prediction task is to know the fundamental mechanism of link formation in particular complex network and using the current topological structural properties to estimate the non-existing links probability. In Fig. 1, solid lines represent links in the network at time *t*, and dashed lines represent the link that may occur in the future (for the sake of clarity only two dotted lines are shown in Fig. 1). Maria and Adam are friends, Maria and Sophia are also friends at time *t*. Possibly Maria introduces Sophia with Adam, and they become friends as well. Similarly, Sophia and David may become friend at time *t*′. A link prediction algorithm awards a similarity score *S*_*xy*_ to all links *l*_*xy*_ ∈ *L* based on some pre-defined criterion. Note that *l*_*xy*_ represents link between nodes *x* and *y*. If *S*_*xy*_ is greater than or equal to a threshold, then a link is predicted between nodes *x* and *y*.

## Literature Review

Considerable body of literature is devoted to the study of link prediction in complex networks (see^7^ and the references therein). The problem is addressed both from graph theory, and machine learning perspective. Due to space limitation, we cannot elaborate on the research work in the domain of machine learning. The reader is referred to Nickle *et al*.^17^ and Wang *et al*.^18^. In the following, we summarize the important works based on graph theoretic approach.

Wang *et al*.^19^ presented a popularity based structural perturbation algorithm that made use of current popularity of node based on the assumption that an active node has more affinity to attract future nodes. The algorithm is based on similarity based approach that measures the possibility of links through knowing collective aspects, i.e., common friends, age differences, professions, and tracing locations which the two end points have in common. The proposed algorithm is evaluated on six data sets and against six algorithms. However, no statistical tests were performed to evaluate the significance of the proposed approach. Yang and Zhang^20^, introduced an algorithm based on the common neighbors and distance metric to predict link in a variety of real world networks from the available topological structure of the network. The algorithm aims to find missing link probability between nodes who do not have common neighbors. The proposed algorithm is tested on eight data sets against standard benchmark algorithms using Areas Under the receiver operating characteristic Curve (AUC) as criterion. The algorithms are executed only once, thus increasing the possibility of data snooping bias. Pan *et al*.^21^ critiqued the real life network to be incomplete and noisy, which makes link prediction algorithms hard to apply. The authors presented an algorithmic framework for missing link prediction by accounting for predefined Hamiltonian structures. Using AUC as evaluation criterion, the proposed framework is evaluated on seven data sets. However, like Wang *et al*.^19^, Yang and Zhang^20^, and Pan *et al*.^21^ did not employ any statistical tests to evaluate the significance of the results.

Liao *et al*.^3^ proposed two algorithms to address missing link prediction problem. The first algorithm is based on Pearson correlation coefficient. In the second algorithm, the correlation based method is integrated with resource allocation algorithm. The second algorithm is found to outperform the existing methods. The proposed second scheme is a parametrized algorithm. However, the control parameter can only influence the correlation factor. Similarly, no statistical tests were performed. Ibrahim and Chen^15^ criticized the existing approaches for missing link predictions based on static graph representation. Authors used temporal information, community structure and centrality to predict the formation of new links. Using AUC as evaluating criterion, authors analyzed the performance of their proposed algorithm using real world data sets. One of the significant drawback of the proposed scheme is the high computational cost.

Zhou *et al*.^2^ empirically investigated missing link prediction of nine well known algorithms on six data sets. The results indicated that common neighbor is the best performing algorithm. The authors further proposed a novel algorithm based on resource allocation process, which achieved superior experimental performance than common neighbor algorithm. Murata and Moriyasu^22^ presented an algorithm based on the proximity measures and weights of existing links in a weighted graph to predict possible future interactions in online social networks. The proposed algorithm was evaluated using Yahoo! Chiebukuro. For a detailed survey of link prediction techniques, the readers are referred to Lou and Zhou^7^.

## Proposed Algorithm

Our proposed algorithm is based on two vital properties of nodes, namely the number of common neighbors and their centrality. Common neighbor refers to the common nodes between two nodes. Centrality refers to the prestige that a node enjoys in a network. Since the seminal work of Freeman^23^, centrality is based on two key factors in an undirected graph, namely closeness and betweenness. Intuitively, closeness centrality refers to the average shortest distance between any given two nodes, whereas betweenness centrality is the measure of control a node has, to influence the flow of information/communication among the nodes of a network. A node will have high betweenness centrality, if the shortest path between the various nodes passes through it. In this work, we consider closeness centrality as parameter for missing link prediction. Formally, we define closeness centrality *C*_*xy*_ between two nodes *x* and *y* in a network with *N* nodes as follows;

$${C}_{xy}=\frac{N}{{d}_{xy}}$$

Note that *d*_*xy*_ is the shortest distance between the nodes *x* and *y*. Using common neighbor, and closeness centrality, we propose a new algorithm for missing link prediction. The algorithm calculates similarity score *S*_*xy*_ as follows;

1 $${S}_{xy}=\alpha \cdot (|\Gamma (x){\cap }^{}\Gamma (y)|)+(1-\alpha )\cdot \frac{N}{{d}_{xy}}$$

Parameter *α* ∈ [0, 1] is a user defined value that controls the weight/importance of common neighbor and centrality. Γ(*x*) represents the neighbors of a node *x*. Note that the value of associated with common neighbor and centrality constitutes a zero sum condition, i.e., increasing the importance of one factor will result in lowering the importance (weight) of other factor.

## Experimental Setting

### Methodology

**Figure 2:**
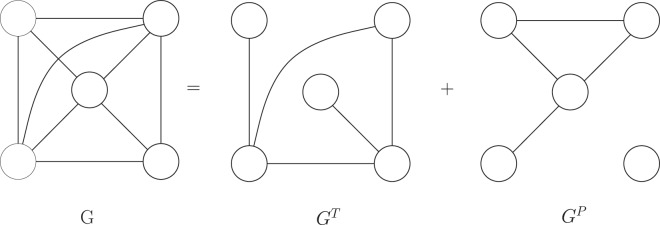
Dividing the original graph in training and probe set.

For investigating the performance of our proposed algorithm, we evaluate the algorithm on eight data sets, and against eight different algorithms. The adopted methodology is as following;

Each data set is divided into two distinct and non-overlapping graphs namely training (*G*^*T*^) and probe (*G*^*P*^) graphs. Training graph (*G*^*T*^) is obtained by randomly sampling over the original graph *G*. The remaining edges, those not included in *G*^*T*^, forms *G*^*P*^. Analogously, the set of edges included in *G*^*T*^ are referred to as *E*^*T*^, and those included in *G*^*P*^ are referred to as *E*^*P*^, i.e., *E* = *E*^*T*^ + *E*^*P*^. Note that *E*^*T*^ and *E*^*P*^ are mutually exclusive. However, the nodes in *G*^*T*^ and *G*^*P*^ may overlap. For our experiments, we have included 80% of edges in *E*^*T*^, and the remaining 20% in *E*^*P*^. Figure 2 is a graphical depiction of the process.

Graph *G*^*T*^ (analogously *E*^*T*^) is the input to a link prediction algorithm, which considers the existing topological properties of the *G*^*T*^, and predicts future links in the form of new graph *G*′. In order to measure the performance of algorithm, we compute the number of true positive (*TP*) edges and false positive (*FP*) edges predicted by an algorithm. An edge *e* is said to be *TP*, if *e* ∈ *G*′, and *e* ∈ *G*^*P*^, i.e., the link is present both in the predicted graph and the probe graph. Recall that edges in training graph and probe graph are mutually exclusive, so it is not possible for an edge to be present simultaneously in *G*^*T*^ and *G*^*P*^. *FP* is defined as *e* ∈ *G*′, and *e* ∉ *G*, i.e., *FP* refers to a wrongly predicted edge which should not exist.

As graph *G*^*T*^ (and hence *G*^*P*^) is obtained randomly, we performed the experiments 15 times to ensure that results obtained are not by chance. For each run, we produced *G*^*T*^ (and hence *G*^*P*^) randomly, *G*^*T*^ was then used as input to the algorithm, which produced the resultant graph *G*′. *G*′ was compared with *G*^*P*^ and *G* to obtain *TP* and *FP*. Our results are based on the average values of the 15 runs. The value of parameter *α* can range from 0 to 1 (both inclusive). For our proposed algorithm we report the average results obtained for *α* = {0.1, 0.2, 0.3, 0.4, 0.5, 0.6, 0.7, 0.8, 0.9}.

### Algorithms

We compare the performance of our proposed algorithm with the following set of algorithms.

1. Common Neighbor and Distance (*CND*): The algorithm is based on two key structural properties of a complex network, i.e., common neighbor and distance. For any two non-connected nodes *x* and *y*, a score *S*_*xy*_ is calculated as shown in Eq. 2 to reflect the likelihood of link formation between the nodes *x* and *y*^20^. Recall that Γ(*x*) refers to the neighbors of node *x*, *CN*_*xy*_ is the number of common nodes between node *x* and *y*, and *d*_*xy*_ is the distance between and *x* and *y*.
  2 $${S}_{xy}=\{\begin{array}{ll}\frac{C{N}_{xy}+1}{2} & \Gamma (x)\cap \Gamma (y)\ne \varnothing \\ \frac{1}{{d}_{xy}} & {\rm{otherwise}}\end{array}$$
2. Preferential Attachment (*PA*): In Preferential Attachment algorithm, the score *S*_*xy*_ depends on the degree of node *x* and *y* respectively, and is calculated as show in Eq. 3 ^24^. Note that *k*_*x*_ represents the degree of a node *x*.
  3 $${S}_{xy}={k}_{x}\cdot {k}_{y}$$
3. Adamic Adar (*AA*): Adamic Adar is based on the hypothesis that it is more likely that two nodes *x* and *y* are introduced by common neighbors who are more likely to be unpopular in the network. In other words, it is more likely that nodes *x* and *y* will be introduced by a node *i* than node *j*, if the degree of *i* is lower than the degree of *j*. The formula for *S*_*xy*_ is given in Eq. 4. Note that Γ(*x*) refers to the neighbors of node *x*.
  4 $${S}_{xy}=\sum _{z\in \Gamma (x)\cap \Gamma (y)}\,\frac{1}{log{K}_{z}}$$
4. Common Neighbor (*CN*): In Common Neighbor algorithm the score for link prediction is computed by finding the number of common neighbors between two distinct nodes^24^. The formula for *S*_*xy*_ calculation of is given in Eq. 5.
  5 $${S}_{xy}=|\Gamma (x){\cap }^{}\Gamma (y)|$$
5. Sorensen Index (*SI*): In Sorensen algorithm, twice of common nodes is divided by the product of degrees of two distinct nodes for calculation of *S*_*xy*_ ^24^.
  6 $${S}_{xy}=\frac{2|\Gamma (x){\cap }^{}\Gamma (y)|}{{k}_{x}+{k}_{y}}$$
6. Jaccard Index (*JI*): Jaccard Index considers only the common neighbors between the nodes to calculate *S*_*xy*_ as following^25^;
  7 $${S}_{xy}=\frac{|\Gamma (x)\cap \Gamma (y)|}{|\Gamma (x)\cup \Gamma (y)|}$$
7. Resource Allocation (*RA*): Resource Allocation (*RA*) calculates *S*_*xy*_ on the basis of intermittent nodes connecting node *x* and *y*. The similarity index is defined as the amount of resource node *x* receives from node *y* through indirect links. Each intermediate link contributes a unit of resource. *RA* is symmetric, i.e., *RA*(*x*, *y*) = *RA*(*y*, *x*)^26^.
  8 $${S}_{xy}=\sum _{z\in \Gamma (x)\cap \Gamma (y)}\,\frac{1}{{K}_{z}}$$
8. Hub Promoted Index (*HPI*): Hub Promoted Index (*HPI*) is a measure defined as the ratio of common neighbors of nodes *x* and *y* to the minimum of degrees of the nodes^27^. HPI is computed as:

9 $${S}_{xy}=\frac{\Gamma (x)\cap \Gamma (y)}{{\rm{\min }}\,\{{k}_{x},{k}_{y}\}}$$

### Data sets

We are using real-world complex network data sets for evaluation of our proposed algorithm against the selected set of algorithms. Gathering a valid data set is time-consuming and labor-intensive process, as most of the data sets are not available publicly. We selected eight popular real-world data sets for our experiments. A brief description of each data set is as following;

1. Karate: Data set of Zachary Karate club network, which shows the correlation of 34 members of a university Karate club. The data set was first studied by Wayne W. Zachary for over three years from 1970 to 1972 to study the clash arose between instructor and administrator^28^.
2. Dolphins: It is a network investigated by Lusseau *et al*.^29^. The network consists of 62 bottlenose dolphins who lived in Doubtful Sound of New Zealand between 1994 and 2001.
3. Polbook: Books about US politics, compiled by Valdis Krebs. Nodes represent books sold online by amazon.com. The edges represent frequent co-purchasing of books by the same buyer. The unpublished network is available online (Social network analysis software & services for organizations, communities, and their consultants. Retrieved from www.orgnet.com).
4. Word: This is the undirected network of common noun and adjective adjacencies for the novel “David Copperfield”. A node denotes either a noun or an adjective. An edge ties two words that occur in adjacent positions. The network is not bipartite, i.e., there are edges connecting adjectives with adjectives, nouns with nouns and adjectives with nouns^30^.
5. Circuit: Electronic circuits can be seen as system where links are wires, and nodes are electronic parts (like capacitors, transistors, etc.). Circuit data is retrieved from www.weizmann.ac.il/mcb/UriAlon/download/collection-complex-networks).
6. Email: This is a network of e-mail exchanges between members of the Universitat Rovira i Virgili (Tarragona). Nodes represent users, and a link is formed between nodes if there is email communication between them. The data is available at http://deim.urv.cat/alexandre.arenas/data/welcome.htm.
7. USAir: The network of the US air transportation system, which contains 332 airports and 2126 airlines which connects the US around the globe^31^.
8. Neural: This data symbolizes the C. Elegans neural network. Graph is being processed in order to remove repeated edges. (See data set at http://wormwiring.org/).

Table 1 Summarizes key properties of the selected data sets.

**Table 1:** Illustration of properties of eight real world networks. *N*: number of nodes in graph (*G*), *M*: number of edges in *G*, <*d*>: average distance, <*k*>: average degree.

Network	*N*	*M*	<*d*>	<*k*>
Karate	34	78	2.408	4.588
USAir	332	2126	2.7381	12.807
Dolphins	62	159	3.357	5.129
Polbook	105	441	3.079	8.400
Word	112	425	2.536	7.589
Neural	306	2147	2.455	14.0327
Circuit	512	819	6.858	3.199
E-mail	1133	5451	3.606	9.622

### Evaluation criterion

A link prediction algorithm assigns a score *S*_*xy*_ to every missing link (i.e., *U* − *E*^*T*^). The score *S*_*xy*_ quantifies the likelihood of a missing link to be existent in the near future. If *S*_*xy*_ equals or surpasses a threshold value, then link is confirmed and considered to occur in the next temporal unit. *AUC* (Area Under the receiver operating characteristic Curve) is used as an evaluation criterion to judge the performance of our selected set of algorithms on the considered data sets. AUC value reflects the probability that a randomly chosen existing link is given a higher similarity score *S*_*xy*_ than a randomly chosen non-existent link. AUC is calculated by picking an existing (*TP*) and a non-existing (*FP*) link and scores are compared. Among *n* independent observations/comparisons, let *n*_1_ observations/comparisons resulted in a higher score for existing links, and *n*_2_ observations have resulted in same score, then *AUC* is calculated as following^32^;

10 $$AUC=\frac{{n}_{1}+0.5{n}_{2}}{n}$$

A good link prediction algorithm should have an *AUC* value close to 1.

## Results and Discussions

**Figure 3:**
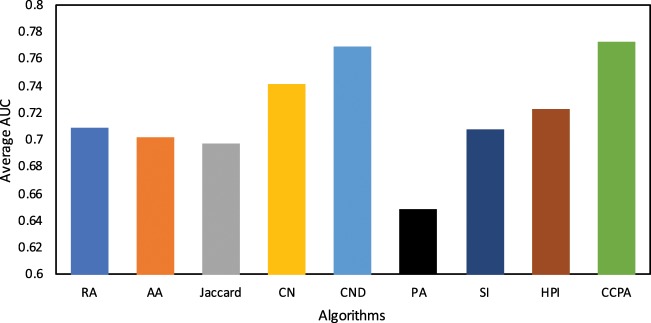
Average AUC of algorithms.

Table 2 summarizes the results based on *AUC* value obtained for each algorithm on various data sets. Standard deviation of *AUC* is also given in the parenthesis. Recall that these results are the average values of 15 runs. Note that for *CCPA*, we consider *α* = {0.1, 0.2, 0.3, 0.4, 0.5, 0.6, 0.7, 0.8, 0.9} and report the average *AUC* over all values of *α* for each data set. We observe that *CCPA*’s average *AUC* values are the highest among the set of algorithms, and thus outperforms all the competing algorithms. The average *AUC* value of *CCPA* is 0.77, which is 7.7% better than the average of *AUC* values of other algorithms. We found that the *AUC* of *CND* (0.76) is very close to that of *CCPA*. However, the performance of *CND* is not consistent. We observed that *CCPA* is the best performing algorithm on 5 data sets (namely USAir, Dolphins, Neural, Circuit, and Email) whereas *CND* is the best performing algorithm on two data sets (Karate and Polbook) only. The worst performing algorithm is *PA* which achieves an average *AUC* value of 0.64, which is 16% less than that of *CCPA*. Figure 3, depicts the average *AUC* of the considered algorithms on all data sets. It is pertinent to mention that our reported *AUC* values are sightly different than those reported in the literature (for instance see Yang and Zhang^20^). There can be multiple reasons to describe the discrepancy. For instance, just like Yang and Zhang^20^, we sampled *G*^*T*^ and *G*^*P*^ randomly. This might result in different training and probe sets which can ultimately results in different *AUC*. However, the differences are not significant.

**Table 2:** Average *AUC* and standard deviation of the algorithms on selected data sets.

	Karate	USAir	Dolphins	Polbook	Word	Neural	Circuit	Email
RA	0.651 (0.09)	0.685 (0.08)	0.713 (0.08)	0.835 (0.09)	0.638 (0.08)	0.819 (0.06)	0.534 (0.02)	0.793 (0.06)
AA	0.645 (0.07)	0.657 (0.12)	0.701 (0.07)	0.826 (0.08)	0.642 (0.04)	0.803 (0.08)	0.537 (0.03)	0.799 (0.07)
Jaccard	0.542 (0.11)	0.849 (0.08)	0.725 (0.08)	0.792 (0.05)	0.583 (0.08)	0.755 (0.05)	0.525 (0.02)	0.807 (0.09)
CN	0.647 (0.06)	0.895 (0.06)	0.731 (0.08)	0.842 (0.07)	0.639 (0.11)	0.817 (0.07)	0.536 (0.03)	0.816 (0.07)
CND	0.66 (0.11)	0.906 (0.05)	0.746 (0.05)	0.874 (0.05)	0.651 (0.08)	0.821 (0.07)	0.629 (0.09)	0.862 (0.04)
PA	0.593 (0.1)	0.789 (0.06)	0.582 (0.11)	0.606 (0.11)	0.664 (0.12)	0.704 (0.09)	0.527 (0.09)	0.717 (0.1)
SI	0.567 (0.09)	0.844 (0.07)	0.732 (0.09)	0.823 (0.07)	0.582 (0.1)	0.749 (0.08)	0.54 (0.01)	0.825 (0.06)
HPI	0.657 (0.12)	0.88 (0.06)	0.721 (0.06)	0.837 (0.07)	0.594 (0.07)	0.761 (0.12)	0.528 (0.04)	0.797 (0.09)
CCPA	0.646 (0.09)	0.91 (0.06)	0.753 (0.09)	0.864 (0.07)	0.657 (0.09)	0.839 (0.08)	0.631 (0.11)	0.875 (0.05)

**Figure 4:**
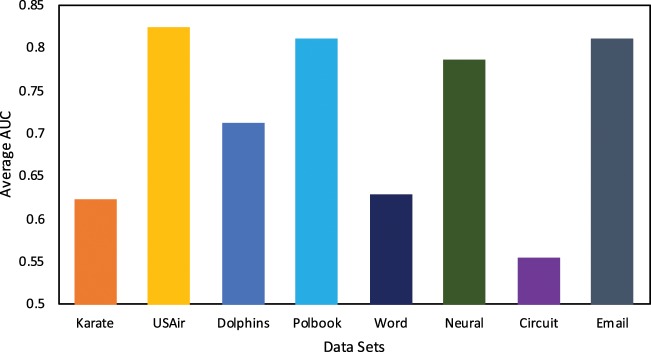
Average *AUC* of algorithms on each data set.

Next, we present the overall performance of the algorithms on the selected data sets. The objective is to identify which data sets are hard to predict in comparison to others. In order to achieve the objective, we calculated the average *AUC* of all the selected set of algorithms on each data set. Results are summarized in Fig. 4. We observed that the worst average *AUC* is achieved by the algorithms on “Circuit” (0.55) and “Word” (0.62) data set, whereas the highest *AUC* is achieved on “US Air” data set (0.823). A circuit graph represents a connection between various parts (such as transistors, capacitors etc). The nodes are represented by capacitors etc, whereas edges are represented by wires. The “Word” data set is a network of adjectives and noun from Charles Dickens novel “David Copperfield”. In the network, nodes represent the adjectives and nouns, whereas edges represent pair of words that occur in adjacent positions in the novel. The low value of *AUC* indicates that it is significantly difficult to predict natural language networks. The highest average *AUC* (0.823) is observed for USAir. US Air is a network of US air transportation, where nodes represent airports, and connection represents flights operated between these air ports by various airlines. As it is a human made network, where connections are not arbitrarily distributed, therefore, predicting the connections are comparatively easier than natural complex networks.

### The effect of choosing *α*

**Figure 5:**
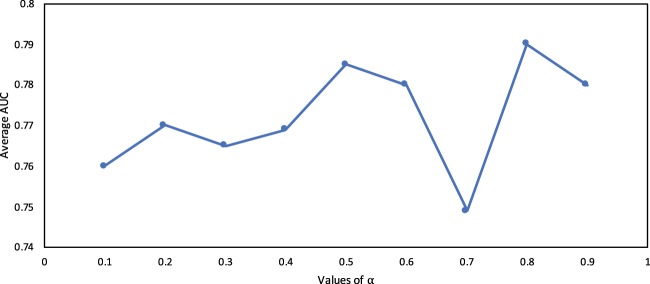
Average *AUC* of *CCPA* for various values of *α*.

In order to find the effect of *α* over the obtained values of *AUC*, we report the results obtained by executing the proposed algorithm on various value of *α* = {0.1, 0.2, 0.3, 0.4, 0.5, 0.6, 0.7, 0.8, 0.9}. We report the average values of *AUC* for a single value of *α* over all the data sets. Figure 5 represents a graphical view of the trend observed in *AUC* for various values of *α*.

It is interesting to note that there is no significant change in the average *AUC* value of *CCPA* based on the change in value of *α*. The minimum average value (0.749) is obtained for *α* = 0.7, whereas the maximum value (0.79) is obtained for *α* = 0.8. The standard deviation of the averaged *AUC* value is 0.013 which is insignificant as well. Even for different data sets, we could not find a trend to identify the value of *α* for which the proposed algorithm *CCPA* will produce optimum results. The optimum value (the value of *α* producing the highest average *AUC* value) varies from data set to data set. For instance, on *Karate* data set the highest *AUC* (0.7) is obtained for *α* = 0.8, whereas for *Dolphins* data set the corresponding *α* value is 0.6. For other data sets, the resultant *α* values are summarized in Table 3. One observation that stands out is that for all data sets the highest average *AUC* value is obtained for *α* ≥ 0.5. For the *Circuit* data set the value is as high as 0.9.

**Table 3:** Best *AUC* values and the corresponding values of *α*.

	Karate	USAir	Dolphins	Polbook	Word	Neural	Circuit	Email
*AUC*	0.7	0.94	0.82	0.9	0.74	0.88	0.68	0.91
*α*	0.8	0.8	0.6	0.5	0.6	0.7	0.9	0.6

In order to improve the applicability of our proposed algorithm in the real world, we attempt to find a statistical property that can identify optimal value of *α*. We analyse the results to find a correlation between the optimal value of *α* for a particular dataset (Table 3) and its key properties (Table 1). For instance, we investigated if there is any correlation between the optimal value of *α* and <*k*> (average degree). We consider various statistical properties (such as <d>, <k>, the ratio of *M* and *N*, clustering coefficient etc), but could not find any correlation that can hold true for all datasets. We then divided the data sets in two classes. *Class 1* includes *Karate*, *Dolphins*, and *Neural* data sets, whereas the remaining 5 data sets are included in *Class 2*. Note that *Class 1* are mainly natural data sets where the relationship between nodes is dependent on a natural phenomenon with little to no human intervention. *Class 2* contains man made networks. For *Class 1* data sets, we identified a correlation between <*d*> (average distance) and optimal value of *α*. We found that smaller values of <*d*> resulted in higher value of *α*. For example, *Karate* data set has the smallest value of <*d*> = 2.408 and the highest optimal value of *α* = 0.8. As the value of <*d*> increases, the optimal value of *α* decreases. Rather surprisingly, we could not establish any correlation between various properties of data sets and optimal value of *α* for *Class 2*. It will be interesting to perform a thorough analysis of the proposed *CCPA* algorithm on a multitude of data sets to obtain a general inference for choosing the optimal value of *α* based on the key statistical properties of a network.

## Conclusion

Motivated by the challenging nature of missing link prediction in complex networks, we present a novel algorithm based on the two key properties of a network, namely common neighbor, and centrality. Unlike previous algorithms, the proposed algorithm is parametrized where user/system has the ability to control the importance of factors under consideration. We compare our proposed algorithm on eight real life data sets and against eight standard algorithms. Results based on *AUC* (Area Under the receiver operating characteristic Curve) shows the superior performance of our proposed algorithm. Further, the performance of algorithm is reviewed with respect to change in the value of *α*.
